# Circular RNA circEIF4G2 aggravates renal fibrosis in diabetic nephropathy by sponging miR‐218

**DOI:** 10.1111/jcmm.16129

**Published:** 2020-12-07

**Authors:** Bojin Xu, Qianqian Wang, Wenyi Li, Lili Xia, Xiaoxu Ge, Lisha Shen, Zhen Cang, Wenfang Peng, Kan Shao, Shan Huang

**Affiliations:** ^1^ Department of Endocrinology Tongren Hospital Affiliated to Shanghai Jiaotong University Shanghai China

**Keywords:** circEIF4G2, diabetic nephropathy, miR‐218, SERBP1

## Abstract

Circular RNAs play essential roles in the development of various human diseases. However, how circRNAs are involved in diabetic nephropathy (DN) are not fully understood. Our study aimed to investigate the effects of circRNA circEIF4G2 on DN. Experiments were performed in the db/db mouse model of type 2 diabetes and NRK‐52E cells. We found that circEIF4G2 was significantly up‐regulated in the kidneys of db/db mice and NRK‐52E cells stimulated by high glucose. circEIF4G2 knockdown inhibited the expressions of TGF‐β1, Collagen I and Fibronectin in high glucose‐stimulated NRK‐52E cells, which could be rescued by miR‐218 inhibitor. Knockdown of SERBP1 reduced the expression of TGF‐β1, Collagen I and Fibronectin in HG‐stimulated NRK‐52E cells. In summary, our findings suggested that circEIF4G2 promotes renal tubular epithelial cell fibrosis via the miR‐218/SERBP1 pathway, presenting a novel insight for DN treatment.

## 1 INTRODUCTION

Diabetic nephropathy (DN), a major microvascular complication of diabetes mellitus, is the leading cause of end‐stage renal disease, representing a global public health concern.^1^ As the cost of treating diabetes and its complications is a significant burden on medical expenditures, it is necessary to identify modifiable factors that contribute to the onset and development of diabetic nephropathy. The main pathological features of diabetic nephropathy include glomerulosclerosis, extracellular matrix deposition and tubulointerstitial fibrosis.^2^ Although controlling blood glucose and blood pressure can slow the progression of diabetic nephropathy, the disease continues to develop gradually. To develop new therapeutic drugs, it is urgent to further understand the molecular mechanisms of DN pathogenesis.

Renal fibrosis is characterized by excessive extracellular matrix synthesis and accumulation, leading to glomerulosclerosis and tubulointerstitial fibrosis. TGF‐β1 is thought to be one of the most important inducers of renal fibrosis.^3^ TGF‐β1 promotes the expression of extracellular matrix proteins, predominantly collagen and fibronectin. Non‐coding RNAs have been shown to regulate renal fibrosis.^4^ LncRNA MALAT1 was significantly increased in mice with streptozocin‐induced diabetic nephropathy.^5^ Erbb4‐IR promotes the progression of renal fibrosis by suppressing the expression of antifibrotic miR29b.^6^ Most studies have focused on the functions of miRNAs and lncRNAs, few explored the roles of circRNAs in diabetic nephropathy. Clarifying the role of circRNA and its target genes is of importance in identifying potential therapeutic targets for DN.

The knockdown of circEIF4G2 suppressed the proliferation, migration and invasion of cervical cancer cells by sponging miR‑218.^7^ CircEIF4G2 was also found to promote the progression of osteosarcoma through sponging miR218.^8^ It is known that miR‐218 plays a key regulatory role in multiple human cancers, while its influence on DN pathology remains uncertain. In this study, we explored the roles of circEIF4G2 in the pathogenesis of DN using the db/db mouse model and NRK‐52E cells. We report that circEIF4G2 promoted renal fibrosis by regulating the miR‐218/SERBP1 pathway, which provides new insights into the pathogenesis of DN.

## 2 MATERIALS AND METHODS

### 2.1 Diabetic nephropathy mouse model

The experimental protocol was approved by the Animal Care and Use Committee of Tongren Hospital Affiliated to Shanghai Jiaotong University. Ten SPF diabetic mice (C57BL/KsJ‐db/db, 8 weeks old) and ten normal control mice (db/m) were purchased from the Model Animal Research Center of Nanjing University. All mice were placed at the animal research facility within the hospital and were maintained in a standard animal house at a 12h light/cycle. The mice were given ad libitum access to food and water.

### 2.2 Cell culture and transfection

The NRK‐52E cells were purchased from the American Type Culture Collection cell bank. NRK‐52E cells were cultured in DMEM complete medium (Hyclone) with 10% FBS (Hyclone) at 37°C in 5% CO^2^. NRK‐52E cells were cultured under normal condition (NC, 5.5 mmol/L D‐glucose and 19.5 mmol/L mannitol) and high glucose conditions (HG, 25 mmol/L D‐glucose) for 24h. Then, the cells were collected for subsequent experiments.

Small interfering RNAs against circEIF4G2 (si‐circEIF4G2), miR‑218 mimic, miR‑218 inhibitor, si‐SERBP1 and corresponding negative controls were designed by Guangzhou RiboBio Co., Ltd. The NRK‐52E cells were transfected with the aforementioned siRNAs alone or in combination. Then, the cells were treated with high glucose to investigate the interactions between circEIF4G2 and miR‐218. Cells were transfected using Lipofectamine 3000 (Invitrogen, Thermo Fisher Scientific, Inc).

### 2.3 RNA extraction and qRT‐PCR

Total RNA of mouse kidney tissue or NRK‐52E cells was extracted using the TRIzol reagent (Invitrogen, Thermo Fisher Scientific) in line with the manufacturer's protocols. Then, RNA (1 μg) was reverse transcribed using the PrimeScriptTM RT Reagent Kit (Takara). PCR was performed on the ABI PRISM 7900HT system (Applied Biosystems). The 2^−DΔCt^ method was adopted to determine the expression of RNAs with GAPDH as the internal reference.

### 2.4 Western blot analysis

The Western blot assay was conducted as previously described.^9^, ^10^ Briefly, total protein of mouse kidney tissue and NRK‐52E cells were extracted with Pierce IP Lysis Buffer (Thermo Fisher Scientific) and were quantified with Pierce BCA Protein Assay Kit (Thermo Scientific). A protein of equal amount (60 μg) was separated by 10% SDS‐PAGE and transferred to PVDF membranes. The membranes were blocked with 5% skim milk and then incubated with primary antibodies against SERBP1 (1:2000, Abcam), TGF‐β1 (1:500, Cell Signaling), Fibronectin and Collagen I (1:200, Abcam). The membranes were incubated at 4°C overnight and were then incubated with horseradish peroxidase‐conjugated secondary antibodies at room temperature for 1 hour. The bands were visualized using the Enhanced Chemiluminescence Kit (GE Healthcare) using the GAPDH antibody as an internal control.

### 2.5 Histopathology

The mice were anesthetized with 3% sodium pentobarbital (1.5 mL/kg). The left kidneys were harvested, fixed in 10% formalin, embedded in paraffin and sectioned at 3 μm. After deparaffinization and rehydration, the sections were stained with haematoxylin and eosin (Sigma Aldrich) according to standard procedures. Representative graphs were captured using a microscope (Olympus). Interstitial injury score was measured in ten randomly selected fields using Image Pro Plus.

### 2.6 Luciferase assay

We used the Starbase 2.0 and TargetScan to predict the potential binding sites between circEIF4G2 and miR‐218. The wild and mutant circEIF4G2 types were synthesized by Shanghai GenePharma and cloned into the pRL‐TK vector (Promega). The NRK‐52E cells were co‐transfected with miR‐218 mimic or mimic‐NC. The luciferase activity was detected 48 hours after transfection using a dual‐luciferase reporter assay system (Promega) with Renilla luciferase as the internal control.

### 2.7 Cell viability assay

Cell proliferation was measured using the Cell Counting Kit‑8 (Dojindo). NRK‐52E cells were seeded into the 96‑well plates (1 x 10^5^/well). After transfection with si‐NC or si‐ circEIF4G2, the optical density was measured at 1, 2, 3, 4 and 5 days at 450 nm wavelength.

### 2.8 Statistical analysis

SPSS version 22.0 (SPSS, IBM) was used for statistical analysis. Data are shown as means and standard deviations (SD). The differences between groups were compared by two‐tailed Student's *t* test or one‐way ANOVA followed by Tukey's post hoc tests. *P* < .05 indicated statistical significance.

## 3 RESULTS

### 3.1 circEIF4G2 is up‐regulated in the db/db mice kidneys

**Figure 1:**
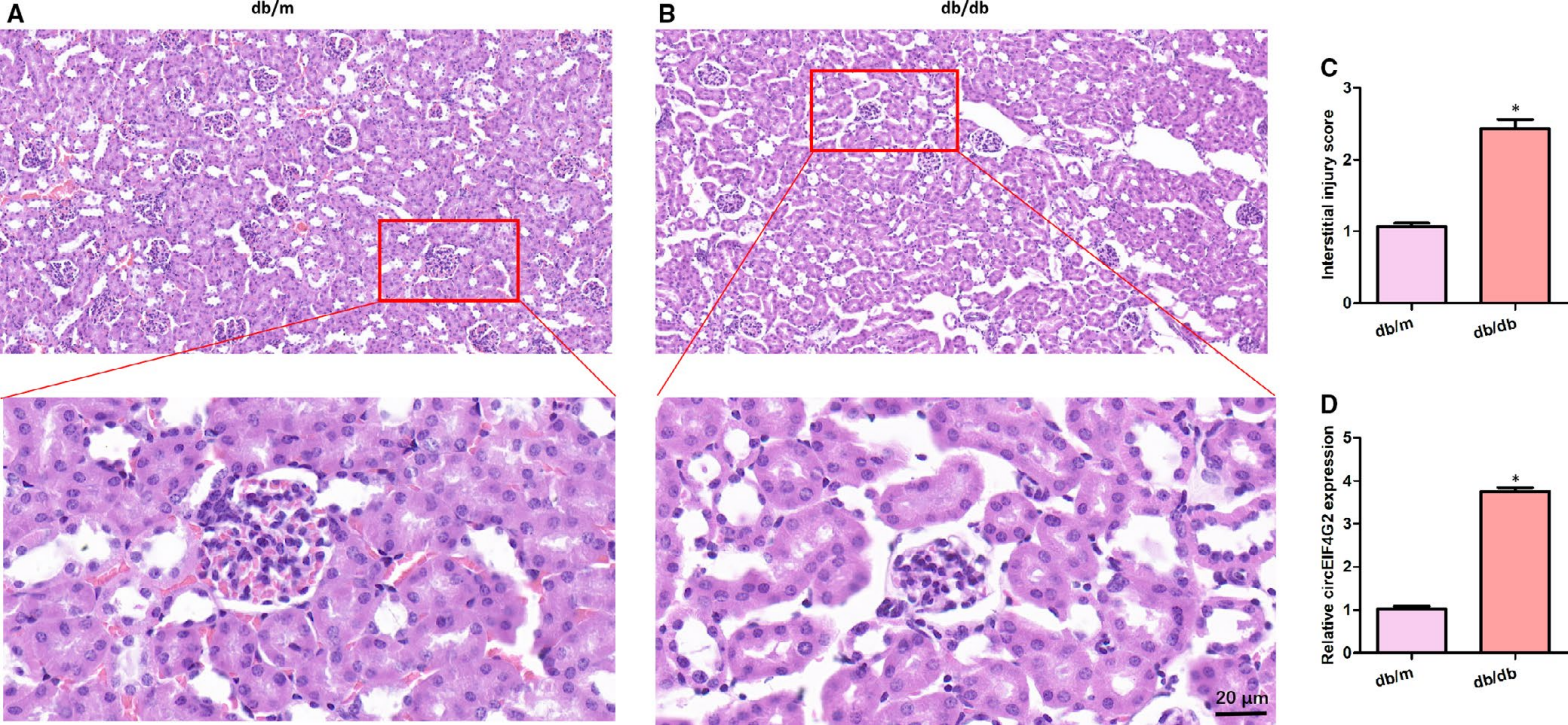
Increased renal fibrosis and downregulation of circEIF4G2 in db/db mice kidney tissues. (A, B) Haematoxylin‐eosin staining was used to detect the degree of the renal lesion in db/db and db/m mice. Scale bar = 20 μm. (C) Interstitial injury score measured using Image Pro Plus. (D) Relative circEIF4G2 expression in mice kidney tissues. Data are shown as means ± SD. *, *P* < .05 [Colour figure can be viewed at wileyonlinelibrary.com]

We first compared the histological structures of kidney tissues between the db/db and the db/m mice. There was obvious pathologic change in the db/db group (Figure 1A,B). The interstitial injury score was significantly higher in the db/db group (Figure 1C). Next, qRT‐PCR was conducted to examine the relative expression of circEIF4G2 in the kidney tissues of db/db DN mice and db/m mice. We found that the expression of circEIF4G2 in the db/db mice kidneys was significantly higher than that of the db/m group. (Figure 1D). These results indicated that circEIF4G2 may be related to the pathogenesis of renal nephropathy in diabetic rats.

### 3.2 circEIF4G2 knockdown inhibits fibrosis in high glucose‐stimulated NRK‐52E cells

**Figure 2:**
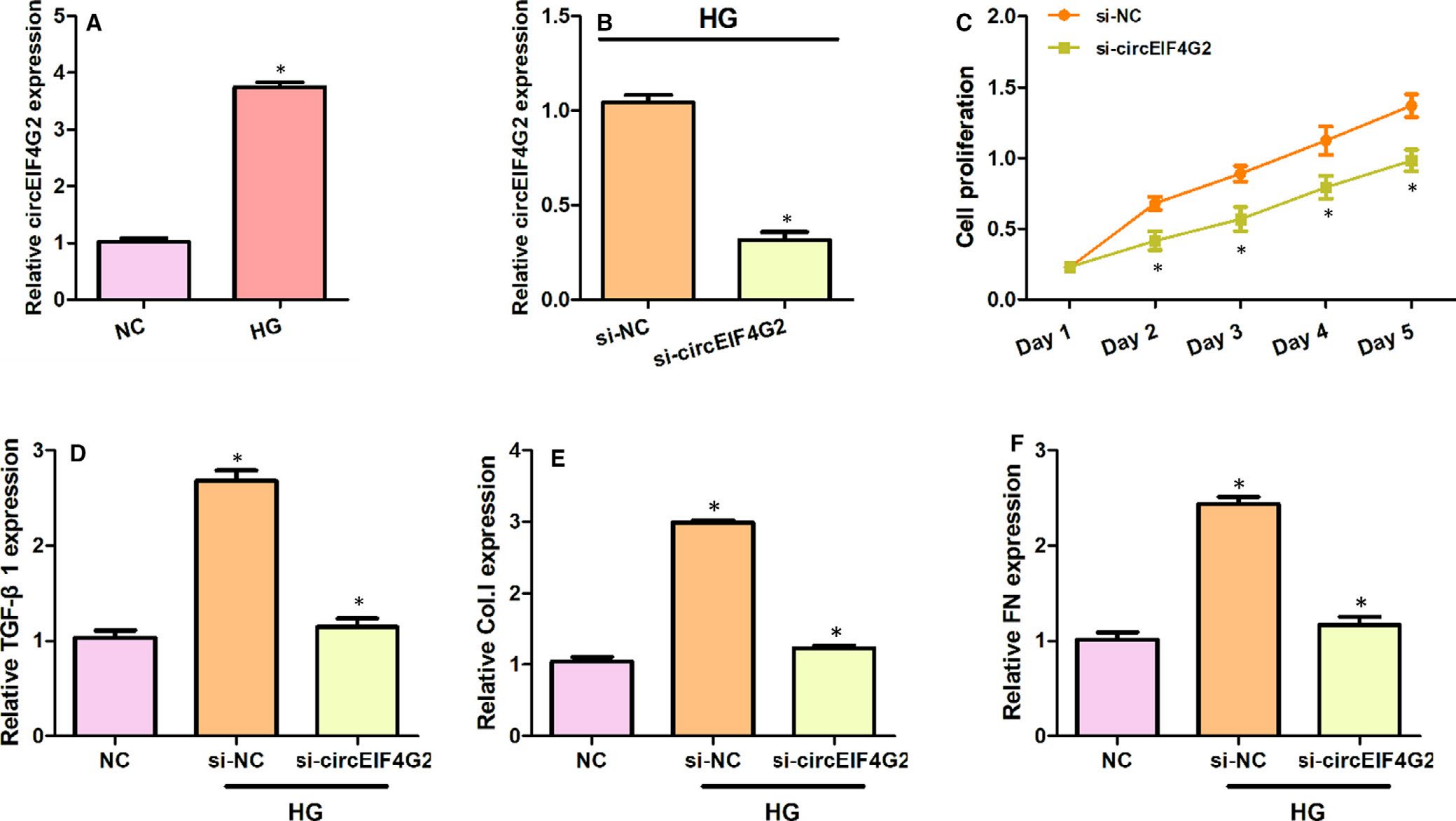
The knockdown of circEIF4G2 inhibits the fibrosis in high glucose‐stimulated NRK‐52E cells. (A) The expression of circEIF4G2 in high glucose‐stimulated NRK‐52E cells. (B) The expression of circEIF4G2 in high glucose‐stimulated NRK‐52E cells transfected with si‐NC and circEIF4G2 siRNA. (C) Cell proliferation was assessed by the CCK‐8 test. (D‐F) The expressions of TGF‐β1, Collagen I and Fibronectin in NRK‐52E cells. Data are shown as means ± SD. *, *P* < .05. Col.I: Collagen I; FN: Fibronectin [Colour figure can be viewed at wileyonlinelibrary.com]

In order to investigate the role of circEIF4G2, we examined the effects of circEIF4G2 knockdown on cell proliferation and the expression of fibrosis‐related markers in NRK‐52E cells. Our results indicated that the relative expression of circEIF4G2 was significantly higher in high glucose‐stimulated NRK‐52E cells than that in the control group (Figure 2A). Following transfection with circEIF4G2 siRNA, the circEIF4G2 expression was significantly reduced in high glucose‐stimulated NRK‐52E cells (Figure 2B).

Using the CCK‐8 assay, we found that the proliferation of NRK‐52E cells was significantly suppressed by circEIF4G2 knockdown (Figure 2C). To explore the effects of circEIF4G2 on renal fibrosis, we detected the expressions of fibrosis‐related biomarkers, including TGF‐β1, Collagen I and Fibronectin, using Western blot. TGF‐β1 promotes renal fibrosis by increasing the production of extracellular matrix proteins (fibronectin and collagen types I, III and IV).^3^, ^11^ We found that high glucose stimulation significantly raised the expressions of TGF‐β1, Collagen I and Fibronectin proteins in NRK‐52E cells, which was restored by circEIF4G2 siRNA (Figure 2D‐F). These results indicated that knockdown of circEIF4G2 inhibited the fibrosis in high glucose‐stimulated NRK‐52E cells.

### 3.3 circEIF4G2 targets miR‐218

**Figure 3:**
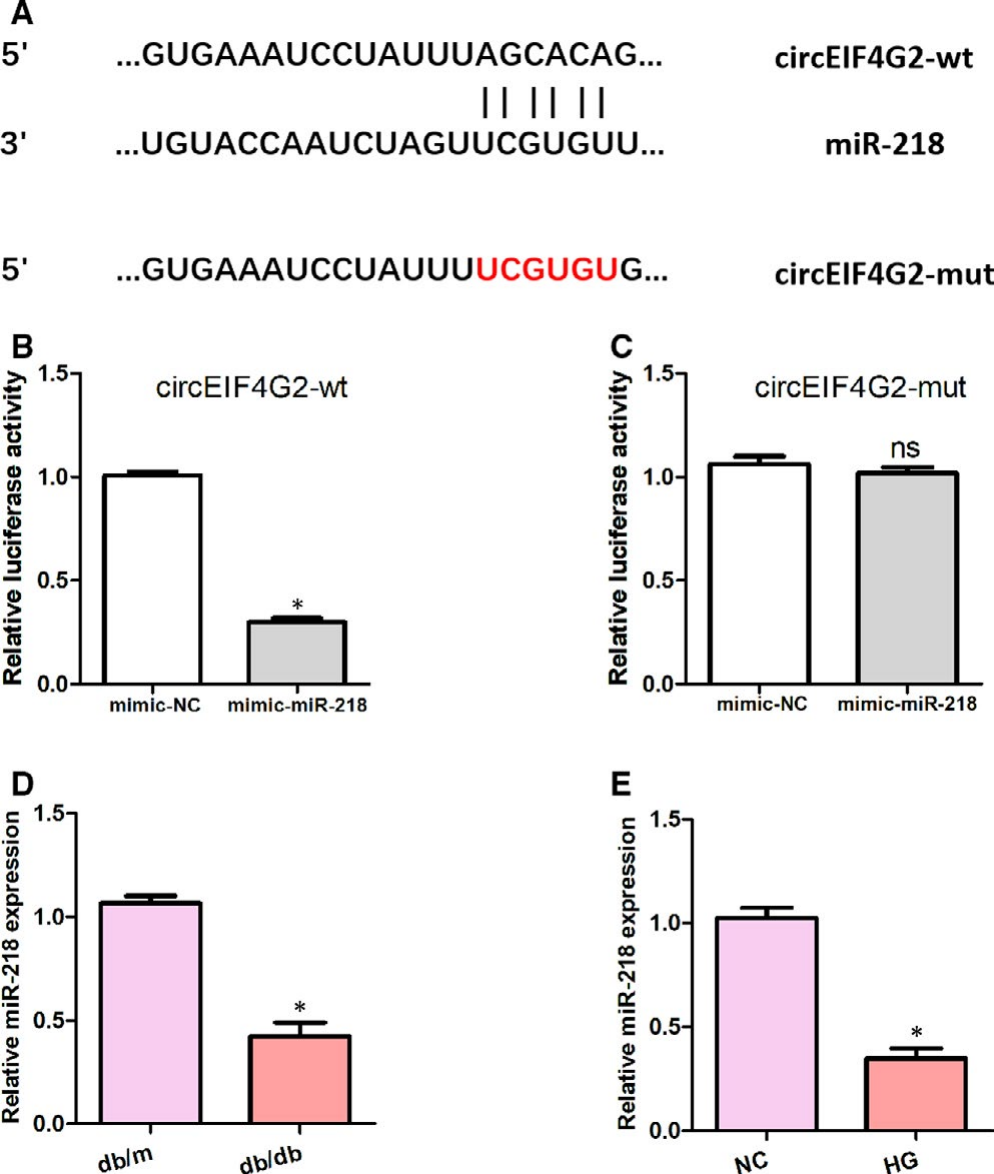
circEIF4G2 inhibits the expression of miR‐218. (A) Predicted binding sites between circEIF4G2 and miR‑218. (C) Luciferase reporter assay was performed to examine the interaction between circEIF4G2 and miR‑218. (D) Expression of miR‑218 in db/db mice kidney tissues. (E) Expression of miR‑218 in high glucose‐stimulated NRK‐52E cells. Data are shown as means ± SD. *, *P* < .05 [Colour figure can be viewed at wileyonlinelibrary.com]

To investigate the mechanisms of the profibrotic effects of circEIF4G2, we predicted the targets of circEIF4G2 by bioinformatics analysis. We identified a potential binding spot for circEIF4G2 in miR‐218 (Figure 3A). Then, we used luciferase activity analysis to establish whether circEIF4G2 targets miR‐218 directly. We observed that the luciferase activity was significantly suppressed in NRK‐52E cells transfected withcircEIF4G2‐wt (Figure 3B) but was not affected in cells transfected with circEIF4G2‐mt (Figure 3C). Furthermore, we observed that the miR‑218 expression was down‐regulated in the kidney tissues of db/db mice (Figure 3D) and high glucose‐stimulated NRK‐52E cells (Figure 3E).

### 3.4 miR‐218 reduces the expression of SERBP1

**Figure 4:**
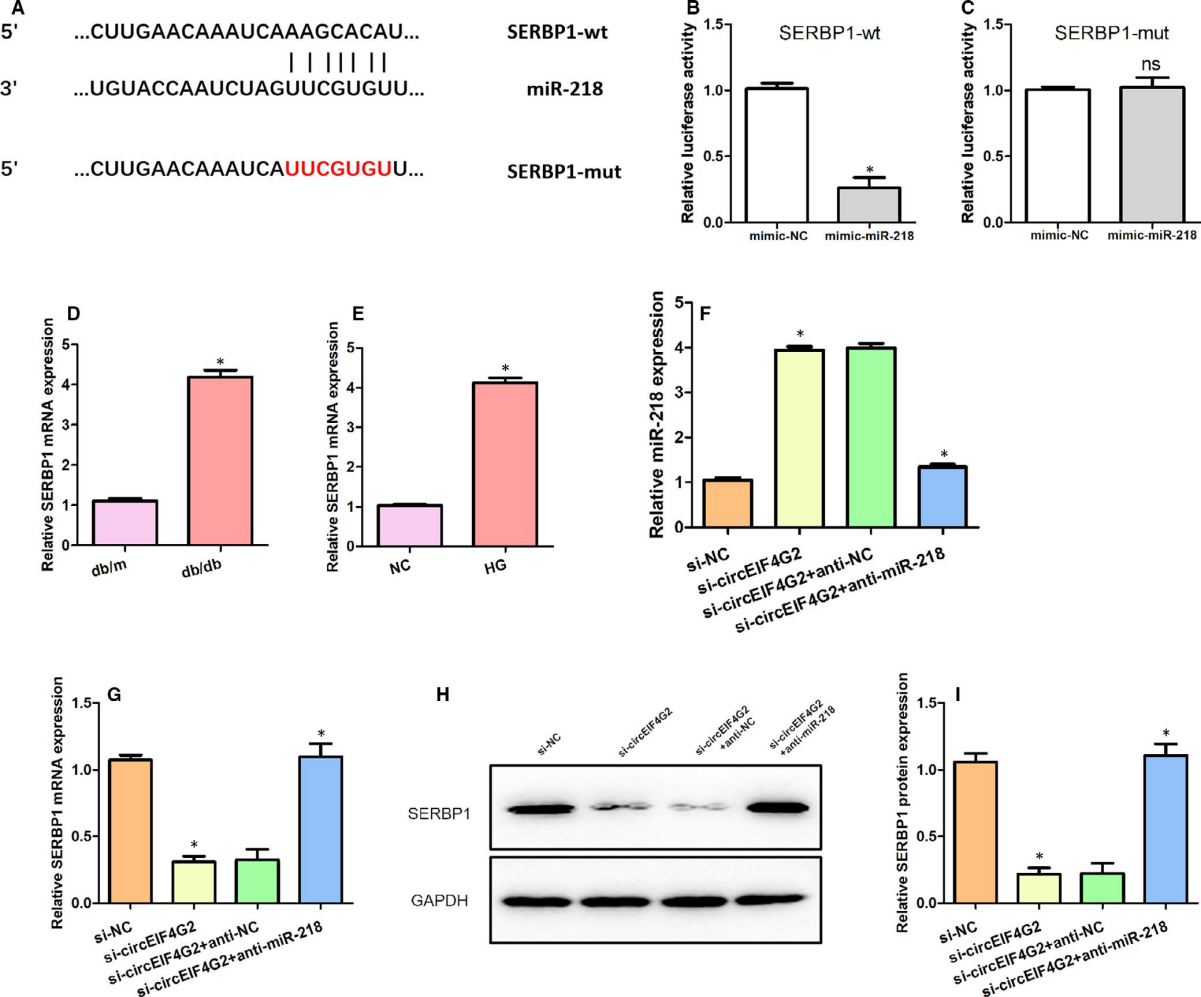
miR‐218 inhibits the expression of SERBP1. (A) Predicted binding sites between miR‐218 and SERBP1. (B) Luciferase activity was significantly decreased in NRK‐52E cells transfected with SERBP1‐wt. (C) Luciferase activity in NRK‐52E cells transfected with SERBP1‐mut. (D) Expression of SERBP1 mRNA in the kidney tissues of db/db mice. (E) Expression of SERBP1 in high glucose‐stimulated NRK‐52E cells. (F) Expression of miR‑218 in high glucose‐stimulated NRK‐52E cells transfected with siNC, si‐circEIF4G2, si‐circEIF4G2 + anti‐NC and si‐circEIF4G2 + anti‐miR‐218. (G) Expression of SERBP1 mRNA in high glucose‐stimulated NRK‐52E cells transfected with siNC, si‐circEIF4G2, si‐circEIF4G2 + anti‐NC and si‐circEIF4G2 + anti‐miR‐218. (H, I) Expression of SERBP1 protein in high glucose‐stimulated NRK‐52E cells transfected with siNC, si‐circEIF4G2, si‐circEIF4G2 + anti‐NC and si‐circEIF4G2 + anti‐miR‐218. Data are shown as means ± SD. *, *P* < .05 [Colour figure can be viewed at wileyonlinelibrary.com]

Using bioinformatics analysis, we found that SERBP1 contains a predicted binding site for miR‐218 (Figure 4A). As shown in Figure 4B,C, when transfected with miR‐218 mimics, the luciferase activity of SERBP1‐wt was significantly decreased compared with the NC group, whereas no significant difference was observed for SERBP1‐mut. These results suggested SERBP1 was a direct target for miR‐218. We observed that the expression of SERBP1 mRNA was markedly higher in the kidney tissues of db/db DN mice (Figure 4D) and high glucose‐stimulated NRK‐52E cells (Figure 4E). To confirm the effects of miR‐218 on SERBP1, we examined the expressions of SERBP1 mRNA and protein in cells transfected with si‐circEIF4G2 and/or miR‐218 inhibitor. Following knockdown of circEIF4G2, the expression of miR‐218 was significantly increased in high glucose‐stimulated NRK‐52E cells, which was counteracted by co‐transfection with the miR‐218 inhibitor (Figure 4F). In contrast, the expression of SERBP1 mRNA was significantly decreased after circEIF4G2 knockdown, which was restored by co‐transfection with the miR‐218 inhibitor (Figure 4G). The same trends were observed for the expression of SERBP1 protein(Figure 4H,I). These findings suggested that SERBP1 was the direct target for miR‐218.

### 3.5 circEIF4G2 knockdown inhibits fibrosis in high glucose‐stimulated NRK‐52E cells via miR‐218/SERBP1

**Figure 5:**
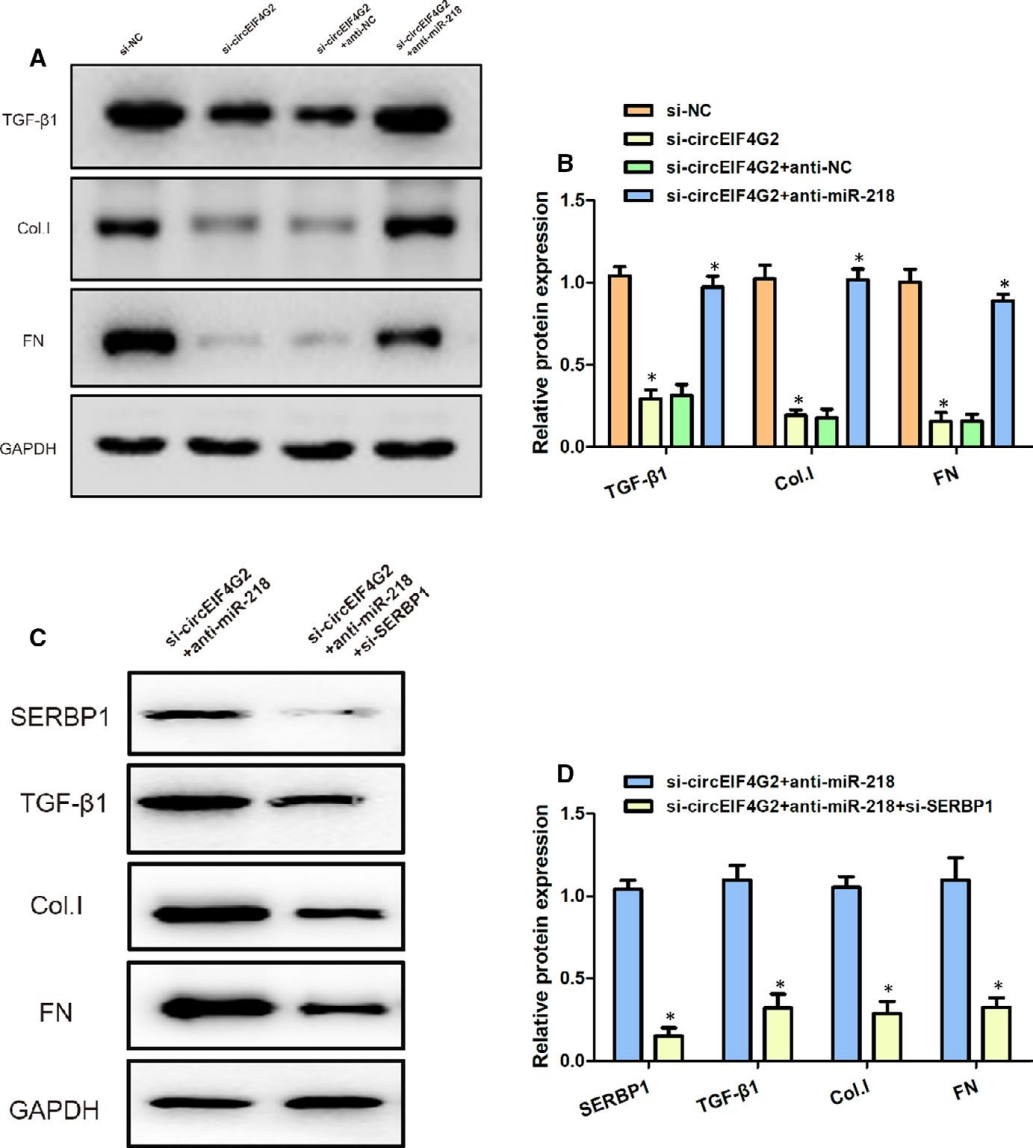
circEIF4G2 inhibits fibrosis in high glucose‐stimulated NRK‐52E cells via miR‐218/SERBP1. (A, B) Expressions of TGF‐β1, Collagen I and Fibronectin in high glucose‐stimulated NRK‐52E cells transfected with siNC, si‐circEIF4G2, si‐circEIF4G2 + anti‐NC and si‐circEIF4G2 + anti‐miR‐218. (C, D) Expressions of SERBP1, TGF‐β1, Collagen I and Fibronectin in high glucose‐stimulated NRK‐52E cells transfected with si‐circEIF4G2 + anti‐miR‐218 and si‐circEIF4G2 + anti‐miR‐218 + si‐SERBP1. Data are shown as means ± SD. *, *P* < .05 [Colour figure can be viewed at wileyonlinelibrary.com]

Finally, we assessed the effects of miR‐218 and SERBP1 on renal fibrosis. The expressions of fibrosis‐related markers, including TGF‐β1, Collagen I and Fibronectin, were evaluated using Western blot. The knockdown of circEIF4G2 cut down the expressions of TGF‐β1, Collagen I and Fibronectin proteins significantly, which were reversed by miR‐218 inhibitor (Figure 5A,B). Moreover, The expressions of TGF‐β1, Collagen I and Fibronectin proteins were markedly decreased by suppressing SERBP1 (Figure 5C,D). These results suggested that circEIF4G2 could regulate the expressions of fibrosis‐related proteins in high glucose‐stimulated NRK‐52E cells via miR‐218/SERBP1.

## 4 DISCUSSION

Previous studies have revealed that circular RNAs are essential regulators of diabetes mellitus (DM) and its related complications. Using the microarray approach, circular RNAs are found to be novel regulators of β‐cell dysfunction via competitively binding miRNAs.^12^ The overexpression of Cdr1as enhanced the proliferation of β‐cells and secretion of insulin.^13^ Peripheral blood circRNA11783‐2 has been related to the risk of coronary artery disease and type 2 diabetes.^14^ CircRNA_010567 was significantly overexpressed in the myocardium of diabetic mice, promoting myocardial fibrosis via the miR‐141/TGF‐β1 pathway.^15^ The expression of circHIPK3 was markedly higher in diabetic retinas and retinal endothelial cells and led to retinal vascular dysfunction by blocking miR‐30a.^16^ CircHIPK3 was also related to the neuropathic pain in diabetic rats.^17^ For diabetic nephropathy, circLRP6 regulated high glucose‐induced mesangial cell proliferation, oxidative damage, extracellular matrix accumulation and inflammatory response by sponging miR‐205.^10^ Knockdown of circRNA_15698 significantly inhibited the production of extracellular matrix‐related proteins by sponging miR‐185.^18^ In our study, we found that circEIF4G2 was significantly overexpressed in the kidneys of db/db mice and high glucose‐stimulated NRK‐52E cells. Furthermore, circEIF4G2 knockdown inhibited the mRNA expression of TGF‐β1, Collagen I and Fibronectin in glucose‐stimulated NRK‐52E cells. Collectively, our findings suggest that the up‐regulation of circEIF4G2 may be related to the fibrosis of diabetic nephropathy.

It has been documented that circRNAs may exert their regulatory capabilities by serving as miRNA sponges. CircRNA_15698 promotes the accumulation of extracellular matrix‐related proteins by blocking miR‐185.^9^ Circular RNA YAP1 was reported to protect the kidney HK‐2 cells from ischaemia/reperfusion injury by acting as a sponge of miR‐21‐5p.^19^ It was shown that miR‐218 plays a key regulator in the progression of multiple human cancers, but its effects on DN remain unclear. It was shown that the expression of miR‐218 was significantly decreased in kidneys with unilateral ureteral obstruction‐induced renal fibrosis.^20^ Our experiments confirmed that the miR‑218 was a direct target for circEIF4G2. The expression of miR‑218 was down‐regulated in both db/db Mice and high glucose‐stimulated NRK‐52E cells. The effects of circEIF4G2 on the expression of fibrosis‐related markers in high glucose‐stimulated NRK‐52E cells could be restored by transfection of miR‐218 inhibitor. These results confirmed that circEIF4G2 regulated the fibrosis of renal tubular epithelial cells by sponging miR‐218.

A previous study reported that miR‐218 is involved in the progression of DN by regulating NF‐κB‐mediated inflammation.^21^ Our results indicated that miR‐218 reduced the expression of TGF‐β1, Collagen I and Fibronectin. But its downstream target genes were unclear. We therefore carried out experiments to elucidate the mechanisms of circEIF4G2 and miR‐218 in renal fibrosis. In our study, SERBP1 was found to be the target of miR‐218, and knockdown of SERBP1 decreased the expression of TGF‐β1, Collagen I and Fibronectin in HG‐stimulated NRK‐52E cells. These results suggested that miR‐218 regulated renal fibrosis via the SERBP1 pathway.

In summary, our findings showed that circEIF4G2 was up‐regulated in the kidney tissues of db/db mice and high glucose‐stimulated NRK‐52E cells. We found that miR‐218 was the target of circEIF4G2 and miR‐218 targeted downstream SERBP1. CircEIF4G2 regulated the production of fibrosis‐related proteins in high glucose‐stimulated NRK‐52E cells via the miR‐218/SERBP1 pathway. Our study provides a novel insight into DN pathogenesis.

## CONFLICT OF INTEREST

The authors declare that they have no conflict of interest.

## AUTHOR CONTRIBUTIONS

**Bojin Xu:** Conceptualization (equal); Data curation (equal); Formal analysis (equal); Writing‐original draft (equal); Writing‐review & editing (equal). **Qianqian Wang:** Conceptualization (equal); Writing‐original draft (equal); Writing‐review & editing (equal). **Wenyi Li:** Conceptualization (equal); Writing‐original draft (equal); Writing‐review & editing (equal). **Lili Xia:** Formal analysis (equal); Investigation (equal). **Xiaoxu Ge:** Investigation (equal). **Lisha Shen:** Investigation (equal). **Zhen Cang:** Investigation (equal). **Wenfang Peng:** Formal analysis (equal); Investigation (equal). **Kan Shao:** Data curation (equal); Formal analysis (equal). **Shan Huang:** Conceptualization (equal); Funding acquisition (equal); Supervision (equal); Writing‐review & editing (equal).

## Data Availability

The data used to support the findings of this study are available from the corresponding author upon request.
